# Prevalence and determinants of Campylobacter infection in under-five children of East Africa: systematic review and meta-analysis

**DOI:** 10.1186/s12879-026-12538-w

**Published:** 2026-01-14

**Authors:** Bruk Abraha, Nega Assefa

**Affiliations:** 1 School of Medical Laboratory Sciences, College of Health and Medical Sciences, Haramaya University, Harar, Ethiopia; 2 College of Veterinary Medicine, Haramaya University, Maya, Ethiopia; 3 College of Health and Medical Sciences, Haramaya University, Harar, Ethiopia

**Keywords:** Age, Animal-exposure, Campylobacter, Meta-analysis, Residence, Sex, Systematic review, Thermophilic

## Abstract

**Background:**

*Campylobacter* species (mainly *C. jejuni* and *C. coli*) are common causes of gastroenteritis in humans, particularly under-fives in middle-income and low-income countries. We conducted a systematic review and meta-analysis of evidence on the prevalence of campylobacteriosis and its association with sociodemographic and animal exposure in under-five children (UFC) of East African countries.

**Methods:**

PubMed/MEDLINE, Wiley Online Library, Research4life programmes (HINARI, AGORA, OARE, and ARDI), Cochrane Library, CINAHL, Google Scholar, and ResearchGate were searched for eligible studies published from 2009 until August 31, 2024, in countries of East Africa. Studies that didn’t present full text and prevalence data in UFC were excluded. Published articles with good quality scores using the JBI (Joanna Briggs Institute) criteria were used for data extraction. The STATA statistical software version 17 (STATACorp, 2021) was used to perform the meta-analysis using a random-effects model. The PROSPERO registration number for this systematic review and meta-analysis is CRD42024599881.

**Results:**

The highest *Campylobacter* prevalence was reported from Ethiopia (63.8%), while the lowest was from Zambia and Madagascar (1.0%). The odds of being positive for thermophilic *Campylobacter* species were 1.2 in males (95% CI = 0.9 - 1.5). Subgroup analysis revealed a pooled OR (odds ratio) of 1.5 (95% CI: 0.9–2.4) in the Horn of Africa and 1.8 (95% CI: 1.2–2.8) in areas situated above 1,500 meters above sea level (m.a.s.l.) for children younger than one year compared to older counterparts. A pooled OR of 1.3 (95% CI = 0.7–2.2) was found for thermophilic *Campylobacter* infection among children exposed to poultry. Eighty percent of studies reported a higher prevalence of campylobacteriosis in the < 2-year age category. Moreover, 63% of studies reported a relatively higher prevalence (*p* > 0.05) in rural settings. The range of prevalence for thermophilic species includes 1.0–19.0% (*C. jejuni*), 0.4 -7.0% (*C. coli*), and 0.9–1.3% (*C. lari*), while no study reported *C. upseliansis.* From these, *C*. *jejuni* accounts for 64–100% of the total *Campylobacter* isolates.

**Conclusion:**

There was a potential trend toward increased risk of thermophilic campylobacteriosis in male children. Elevation and publication year influence the occurrence of thermophilic *Campylobacter* in different age categories. Exposure to poultry is also a potential risk for UFC campylobacteriosis, showing the importance of practicing avoiding/limiting contact with animals.

**Supplementary information:**

The online version contains supplementary material available at 10.1186/s12879-026-12538-w.

## Background

Globally, diarrhea remains the leading cause of child mortality for those aged under five years, causing an annual death of 444,000 children in 2021, which accounts for 9% of all deaths, with the highest deaths recorded in South Asia and sub-Saharan Africa [1]. Globally, the disease is the third leading cause of death in children 1–59 months of age, with nearly 1.7 billion cases of childhood diarrheal disease every year [2].

The common bacterial causes of diarrhea among UFC include *Aeromonas*, *Campylobacter, Cholera, Clostridium difficile*, *Escherichia coli, Salmonella,* and *Shigella* species [2, 3]. Of these bacterial agents, *Campylobacter* accounts for 6.2% of deaths due to diarrhea from 1990 to 2015 estimates. In Ethiopia, Kenya, and Somalia, it is responsible for 1341, 514, and 347 deaths, accounting for 9.2, 5.8, and 3.8%, respectively [3]. In addition, considering all-age deaths, *Campylobacter* is reported to be the responsible pathogen for 123, 000 (95% UI 39, 300–266, 000) deaths in 2019 [4].

Of the 17 species and 6 subspecies assigned to the genus *Campylobacter*, the most often reported clinically important species in humans are *C. jejuni* (subspecies *jejuni*) and *C. coli*. Other species, such as *C. lari* and *C. upsaliensis,* have also been reported in diarrhea cases [5]. These thermophilic species can grow at 42 °C and mainly inhabit animals. In adult animals, they do not normally cause clinical disease yet serve as a covert source of infection in humans [6, 7]. *C. jejuni* typically accounts for 90% of human gastroenteritis cases in the United Kingdom and the United States, while *C. coli* accounting for the remaining ~10%. Also, most human strains were identified as commensal gastrointestinal inhabitants of domesticated animals [8]. In 2022, the first most reported zoonosis in humans in European Union countries was campylobacteriosis, in which the majority of cases were due to thermophilic species such as *C. jejuni* (87.6%), *C. coli* (10.7%), *C. upsaliensis* (0.17%) and *C. lari* (0.12%), with *C. fetus* and other species accounting for 0.26% and 1.1%, respectively [9].

Animal and environmental sources of infection for campylobacteriosis include domestic fowl ( > 50% human cases), other birds (reservoir), mammals (cattle, sheep, pig, dog, cat), oysters, crabs, and surface water (contaminated by birds). The transmission mode is through eating or drinking raw meat, raw milk, fresh cheese, and water, or through direct contact [6, 10]. Diarrheic pets were also reported as a source of infection with multidrug-resistant strains [11]. In Asian, South American, and African countries, several studies have shown that children’s campylobacteriosis is associated with exposure to animals or their products [12–16].

Previous systematic reviews have reported on the prevalence of thermophilic *Campylobacter* in animals and humans of all age categories in sub-Saharan African countries based on studies reported from 1997 to 2018, but did not address the determinants for campylobacteriosis [17]. Particularly, from Ethiopia, an attempt was made to conduct a systematic review and meta-analysis of *Campylobacter* in humans, but regardless of the children’s clinical status and age (i.e., including > five years old) [18]. These showed limited availability of aggregate data on thermophilic *Campylobacter* species among diarrheic UFC in East Africa. Therefore, the objective of this review was to provide a pooled estimate on the prevalence of *Campylobacter* in UFC using studies in East Africa, with particular emphasis on the association of children’s sociodemographic and animal exposure status with thermophilic *Campylobacter* in diarrheic groups.

## Methods

### Study protocol

The Preferred Reporting Items for Systematic Reviews and Meta-analysis (PRISMA) guideline was used to report the findings of this review [19]. The PRISMA checklist was also strictly followed while conducting this systematic review.

### Selection of studies

#### Eligibility criteria

The acronym “PECOS” was used to consider the eligibility of studies for this review as follows:

P = Population (diarrheic/non-diarrheic UFC in East Africa)

E = Exposure (risk factors such as sex, age, residence, contact with animals)

C = Comparison (not exposed to the mentioned risk factors)

O = Outcomes (presence of *Campylobacter*)

S = Study design (observational studies reporting prevalence)

#### Selection strategy

Materials identified from the electronic databases were exported to EndNote reference software version 21 (Clarivate, Philadelphia, PA), and duplicated studies were removed instantly. Then, two independent reviewers screened the titles, abstracts, and full texts. Finally, eligible studies were appraised for overall validity (internal and external) using the STROBE (Strengthening the Reporting of Observational Studies in Epidemiology) statement for observational studies by two independent reviewers. The JBI criteria were used to score articles. Discrepancies between the reviewers in the judgment of whether to include further evaluation were solved by discussion and consensus.

#### Inclusion and exclusion criteria

In the initial screening/evaluation of titles, abstracts, and full texts, there were sets of predefined inclusion and exclusion criteria for eligibility to be included for final evaluation. Published or grey literature articles obtained through ancestry searches or in response to email requests and published until August 31, 2024, were included in this review.

**Fig. 1:**
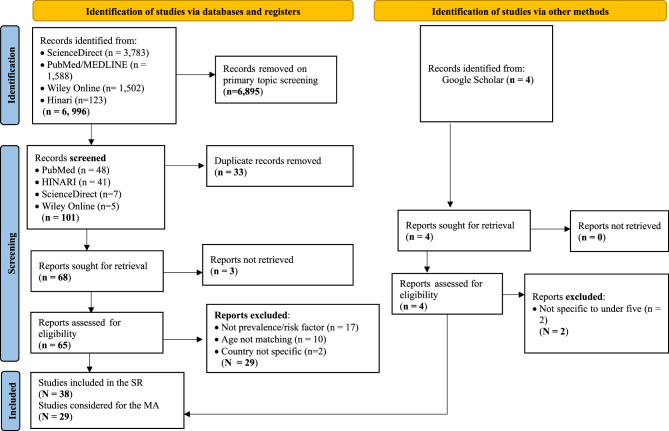
PRISMA flow diagram of literature search and selection criteria (the flow diagram is adapted from Page et al. [19])

All observational studies reporting the prevalence and associated risk factors for *Campylobacter* or thermophilic *Campylobacter* species (*C*. *jejuni*, *C. coli*, *C. lari*, and *C. upsaliensis*) in under-five diarrheic/non-diarrheic children of countries in East Africa (Burundi, Comoros, Djibouti, Eritrea, Ethiopia, Kenya, Madagascar, Malawi, Mauritius, Mayotte, Mozambique, Réunion, Rwanda, Socotra, South Sudan, Seychelles, Somalia, Tanzania, Uganda, Zambia, and Zimbabwe) attending health care facilities as well as at the community level. Materials were considered as a source if they present articles/manuscripts in the public domain. Any study conducted outside of the countries in East Africa was excluded during the first screening. Articles that didn’t present full text, studies with unclear descriptions of methods, studies conducted from stored samples, outbreak cases, and incomplete data (after checking their supplementary materials) were excluded after contacting the primary author through email and the ResearchGate platform. In addition, duplicate publications were excluded. For the meta-analysis, studies were excluded if conducted in community settings and with mixed study populations (non-diarrheic and diarrheic children). The article selection and exclusion process are shown in Fig. 1.

### Searching strategy

Advanced search strategies were applied in major databases to retrieve articles closely related to campylobacteriosis in enteric illness of children. The search strategy aims to find published studies, relevant reviews/meta-analyses, and theses. Electronic databases such as PubMed/MEDLINE, ScienceDirect, WileyOnline Library, Research4life programmes (HINARI, AGORA, OARE, and ARDI), Cochrane Library, CINAHL, Google Scholar, and ResearchGate were searched for any relevant materials. In addition, websites of professional organizations (WHO, CDC, WOAH) were also searched for any material published outside of peer-reviewed journals. Manual searches were performed to identify eligible grey literature (publications, abstracts, and conference proceedings) available in Opengrey (http://www.opengrey.eu). The search was restricted to the English language until August 31, 2024.

The steps in the searching strategy include an initial limited search of the text words contained in the title, abstract, and index terms of the article. Then, a search was conducted for all identified keywords and index terms in all the databases. Subject headings relevant to each database were used, for example, MeSH (medical subject headings) for PubMed/MEDLINE and subject terms for Research4life programmes. Finally, the reference list of all identified studies was searched for additional studies.

The initial keywords used for searching include “*Campylobacter*”, “*C. jejuni/C. coli/C. lari/C. upsaliensis*”, “prevalence”, “under-five”, “diarrhea/diarrhea”, “children”, “risk factors”, and “East Africa” and “specific names of countries in East Africa”. All terms were searched using Boolean operators such as “AND” and “OR”. In PubMed, additional filters applied were humans, child: birth-18 years, newborn: birth-1 month, infant: birth-23 months, infant: 1–23 months, and preschool child: 2–5 years. The search queries used in the different search engines are summarized in Table Supplementary 1. For additional information, after checking supplementary materials, the authors were contacted to obtain full access to articles and clarifications on relevant issues. The protocol was registered with the International Prospective Register of Systematic Reviews (PROSPERO CRD42024599881), which can be fully accessed upon completion.

### Assessment of study quality

Before considering studies for data extraction, two independent reviewers assessed methodological quality using the critical appraisal checklist of the JBI used for studies reporting prevalence data [20]. The checklist is based on nine questions dealing with the quality of a study, and each article receives a value representing the extent to which it met the mentioned questions (Table Supplementary 2). The values were “yes”, “no”, “unclear”, and “not applicable”. Then, a study was considered for final acceptance if the score was ≥ 70%. The overall objective is to ensure the study’s overall validity. Disagreements were resolved by consensus.

### Data extraction

Full texts of articles that were categorized as included were extracted systematically by using a standardized data extraction tool as recommended by the JBI [21]. Descriptive and quantitative data from each eligible study were extracted by two independent reviewers. The domains used for extraction include outcomes, methods used to obtain the outcome (isolation and identification of *Campylobacter*), setting (health care facility, community-based), geographical location (e.g., Greater Horn of Africa), average elevation (above or below 1,500 m.a.s.l.), study design, children’s attributes, and animal exposure status. The relevant data was entered into Microsoft Excel 2021. Finally, the data includes first author, publication year, data collection period, country, type of observational study design, study setting, children’s age, children’s gender/sex, children’s residence, children’s exposure status to animals, types of specimens, laboratory detection method, total number of children recruited into the study (study size), events of *Campylobacter,* events of thermophilic *Campylobacter*, events of *C. jejuni/coli,* events of *C. jejuni,* and events of *C. coli.* Djibouti, Ethiopia, Kenya, Somalia, South Sudan, and Uganda were considered part of the Greater Horn of Africa.

### Statistical analysis

The STATA statistical software version 17 [22] was used to perform the meta-analysis. The effect sizes (proportion and OR) were pooled using the random-effects model [23, 24] at a 95% confidence level. The heterogeneity of studies was determined using I^2^ statistics, with 50% as a cut-off value for deciding a high level of heterogeneity. Moreover, the Galbraith plot was used to detect the study-specific effect sizes with potential outliers to assess the heterogeneity among the effect sizes. To determine the source of heterogeneity, a subgroup meta-analysis and meta-regression test were conducted, with a p-value of < 0.05 used as the cut-off value for significance. The presence of publication bias was assessed by both visual inspection of funnel plots and Egger’s tests [25]. The leave-one-out meta-analysis was performed to investigate the influence of each study on the overall effect size estimate. A forest plot was used to provide point estimates and 95% confidence intervals.

### Outcome measurements

The primary outcome measures are the pooled estimate of *Campylobacter* occurrence in any UFC and the association of a child’s sociodemographic attributes and animal exposure status with thermophilic *Campylobacter* in diarrheic children attending health care facilities. The secondary outcome is the proportion of specific thermophilic *Campylobacter* species in diarrheic UFC in health care settings.

### Assessment of bias

Two independent reviewers assessed the risk of bias using the AHRQ (Agency for Healthcare Research and Quality) criteria for Assessing the Risk of Bias of Individual Studies in Systematic Reviews of Health Care Interventions [26], which is based on sets of nine questions for cross-sectional and 11 for case-control (Table Supplementary 3). The risk of bias was categorized as “high” when the study had a “yes” score of less than 49%, as “moderate” when the study had a “yes” score from 50% to 69%, and as “low” when the study had a “yes” score of more than 70% for bias risk questions. To decide on the confidence of evidence, we considered the adequacy of sample size, coverage of the identified sample size (response rate), validity of methods used for identification of the bacteria, and appropriateness of the statistical analysis.

## Results

### Search results

Our literature search of PubMed/MEDLINE, Research4life programmes, ScienceDirect, and Wiley online displayed 6996 records, and 6895 were removed on primary topic screening. Thus, 101 records from the databases and an additional four articles from Google Scholar, totaling 105, were screened for the abstract. Searching the Cochrane Library and CINHAL didn’t yield articles related to the topic. Hand searches of reference lists and websites of relevant organizations did not produce any additional articles that fulfilled the inclusion criteria. Of the 105 articles, 33 duplicate articles were removed with the help of EndNote and manual tracing, while three were unable to access their full text. Thus, 69 articles were screened using a full-text record, and 31 articles were excluded as they lacked age specification, failed to report prevalence, or didn’t specify a country. Finally, 38 articles passed the eligibility criteria and quality assessment (Fig. 1; Table Supplementary 4). The smallest score for quality assessment was 77% according to the JBI critical appraisal checklist used for studies reporting prevalence data. For the meta-analysis, we excluded nine studies that were conducted in community settings and had mixed study populations (both non-diarrheic and diarrheic children). Thus, only 29 studies were considered for meta-analysis of campylobacteriosis in diarrheic children from health care-based studies (Table 1).

**Table 1:** The summary of the study’s characteristics and reported *Campylobacter* outcome and associated risk factors (sociodemographic and animal exposure) in under-five children of the East Africa region from 2009 to 2024 for systematic review and meta-analysis

Author (year)	Study period	Country	Age	Design	Setting	Detection method	N	№ of events	Prevalence in % (95% CI)	Risk factors identified
Abay et al. (2024) [27]	NS	Ethiopia	UFC*	CS	HC	C, LA	214	14	6.5 (3.6–10.7)	Sex, age, residence, animal exposure
Behailu et al. (2022) [28]	2021	Ethiopia	UFC*	CS	HC	C, BC	235	16	6.8 (3.9–10.8)	Sex, age, residence, animal exposure
Belina et al. (2023) [29]	2021/23	Ethiopia	UFC*	CS	HC	C, BC	262	22	8.4 (5.3–12.4)	Residence
Mshana et al. (2009) [30]	2005	Uganda	UFC*	CS	HC	C, BC	226	21	9.3 (5.8–13.9)	Age
Chercos et al. (2024) [31]	2017/19	Tanzania	UFC*	CA	HC	PCR	313	34	10.9 (7.6–14.8)	Sex, age
Chiyangi et al. (2017) [32]	2016	Zambia	UFC*	CS	HC	C, BC	271	3	1.1 (0.2–3.2)	Sex
Chuma et al. (2016) [33]	2006/07	Tanzania	UFC*	CS	HC	PCR	268	51	19.0 (14.5–24.2)	Sex, age, animal exposure
Deogratias et al. (2014) [34]	2012/13	Tanzania	UFC*	CS	HC	C, BC	300	29	9.7 (6.6–13.6)	Sex, age, animal exposure
Lengerh et al. (2013) [35]	2011/12	Ethiopia	UFC*	CS	HC	C, LA	281	44	15.7 (11.6–20.4)	Sex, age, residence, animal exposure
Getamesay et al. (2014) [36]	2011	Ethiopia	UFC*	CS	HC	C, BC	158	20	12.7 (7.9–18.9)	Sex, age
Mulu et al. (2024) [37]	2021/22	Ethiopia	UFC*	CS	HC	C, MALDI	303	59	19.5 (15.2–24.4)	Sex, age
Nigusu et al. (2022) [38]	2020	Ethiopia	UFC*	CS	HC	C, BC	214	19	8.9 (5.4–13.5)	Age, residence, animal exposure
Tafa et al. (2014) [39]	2012	Ethiopia	UFC*	CS	HC	C, BC	227	38	16.7 (12.1–22.2)	Sex, age, animal exposure
Worku et al. (2024) [40]	2020/23	Ethiopia	UFC*	CS	HC	C, MALDI	428	30	7.0 (4.8–9.9)	Sex, age, residence, animal exposure
Zachariah et al. (2021) [41]	2021	Kenya	UFC*	CS	HC	C, BC	139	18	12.9 (7.8–19.7)	Sex, age
Ouko et al. (2021) [42]	2017/19	Kenya	UFC*	CS	HC	C, PCR	977	118	12.1 (10.1–14.3)	Sex, age, residence, animal exposure
Mason et al. (2013) [43]	1997/07	Malawi	UFC*	CS	HC	PCR	1941	415	21.4 (19.6–23.3)	Age
Conan et al. (2017) [44]	2008/11	Kenya	UFC*	CA	HC	C, PCR	73	14	19.2 (10.9–30.1)	Animal exposure
Oketcho et al. (2012) [45]	2011	Tanzania	6–59*	CS	HC	C, BC	151	5	3.3 (1.1–7.6)	–
Beatty et al. (2009) [46]	2001/03	Kenya	UFC*	CS	HC	C, BC	2550	417	16.4 (14.9–17.8)	Residence
O’Reilly et al. (2012) [47]	2005/07	Kenya	UFC*	CS	HC	C, BC	1146	57	4.9 (3.8–6.4)	
Gosselin et al. (2017) [48]	2009/11	Tanzania	UFC*	CO	HC	PCR	123	3	2.4 (0.5–6.9)	–
Tickell et al. (2017) [49]	2011/14	Kenya	6–59*	CS	HC	C, BC	1355	100	7.4 (6.0–8.9)	–
Mushi et al. (2014) [50]	2012/13	Tanzania	24*	CS	HC	C, BC	300	14	4.7 (2.6–7.7)	–
Randremanana et al. (2016) [51]	2011/14	Madagascar	UFC*	CA	HC	C, LA	199	2	1.0 (0.1–3.6)	–
Kabayiza et al. (2014) [52]	2009/12	Rwanda	UFC*	CS	HC	PCR	800	147	18.4 (15.7–21.2)	
van Eijk et al. (2010) [53]	1997/01	Kenya	24 m*	CO	HC	C, BC	513	114	22.2 (18.7–26.1)	–
Chisenga et al. (2018) [54]	2012/13	Zambia	UFC*	CS	HC	PCR	1599	459	28.7 (26.5–30.9)	–
Hugho et al. (2023) [55]	2020/22	Tanzania	UFC*	CS	HC	PCR	146	5	3.4 (1.1–7.8)	Age
Randremanana et al. (2012) [56]	2008/09	Madagascar	UFC*	CS	COM	C, LA	2196	213	9.7 (8.5–11.0)	–
Platts-Mills et al. (2014) [57]	2013	Tanzania	12*	CO	COM	PCR	69	27	39.1 (27.6–51.6)	–
McQuade et al. (2020) [58]	2015	Zimbabwe	18†	CO	COM	PCR	721	230	31.9 (28.5–35.4)	–
Terefe et al. (2020) [59]	2018	Ethiopia	12–16†	CS	COM	PCR	100	15	15.0 (8.6–23.5)	–
Kiarie et al. (2023) [16]	2021	Kenya	24†	CS	COM	C, PCR	540	26	4.80 (3.20–6.90)	Animal exposure
Chen et al. (2021) [15]	2018	Ethiopia	12–16†	CS	COM	PCR	101	51	50.1 (40.4–60.6)	Sex, animal exposure
Berendes et al. (2019) [60]	2016	Mozambique	14†	CA	COM	PCR	120	13	10.8 (5.9–17.8)	–
Deblais et al. (2023) [61]	2020/22	Ethiopia	12–14†	CS	COM	PCR	1073	685	63.8 (60.9–66.7)	–
Budge et al. (2020) [62]	2019	Ethiopia	10–18†	CS	COM	C, BC	35	17	48.6 (31.4–66.0)	–

* means diarrheic children only; † indicates population is both diarrheic and non-diarrheic; *UFC = Under-five children; HC = Health care; MALDI = Matrix-Assisted Desorption/Ionization-Time of Flight Mass Spectrometry; CS = Cross-sectional; CA = Case-control; CO = Cohort; NS = Not specified; N = Sample size; C = Culture; BC = Biochemical test; PCR = Polymerase chain reaction; LA = Latex agglutination test*

### Study characteristics

From the 21 countries in East Africa, data on *Campylobacter* in UFC were available from nine (42.9%) countries of the 38 articles (Table 1). The included studies were published between the years 2009 and 2024. From the countries of East Africa, the highest contribution was from Ethiopia (*n* = 13; ~ 34%), followed by Kenya (*n* = 9; ~ 24%), and Tanzania (*n* = 8; ~ 21%). Zambia and Madagascar each account for ~5% (*n* = 2), while Malawi, Zimbabwe, Mozambique, and Rwanda each account for ~3% (*n* = 1).

The majority (76.3%) of the studies were carried out in health center settings, and 78.9% employed a cross-sectional study design. The main detection and identification method was culture on selective media (at 37 to 42 °C) with biochemical tests (*n* = 16; 42.1%), followed by direct PCR (Polymerase Chain Reaction) tests (i.e., without culture) (*n* = 12; 31.6%), culture with PCR (*n* = 5; 13.2%), culture with antigen detection (immune-agglutination) tests (*n* = 4; 10.5%). Two studies applied MALDI-TOF (Matrix-Assisted Laser Desorption/Ionization Time-of-Flight) for final confirmation of *Campylobacter* isolates. The study sizes ranged from 35 to 2550 individuals. Twenty studies (52.6%) considered any UFC individuals regardless of gender and socio-demographic background in their inclusion criteria, while 10 (26.3%) considered only < 24 months. The remaining eight (21%) studies excluded infants aged 1–6 months. Overall, 16 (42.1%), 14 (36.8%), 12 (31.6%), and eight (21.1%) studies presented separate data on the occurrence of *Campylobacter* by age, sex, animal exposure, and residence categories, respectively.

Of the 38 studies, 22 provide data on at least one variable (risk factor) of interest, such as sex, age, residence, and animal exposure (Table 2). Likewise, the majority (*n* = 28; 73.7%) targeted the detection of thermophilic *Campylobacter* in the study population (Table 3).

**Table 2:** Summary of risk factors (sociodemographic and animal exposure) associated with *Campylobacter* outcome in under-five children of East Africa from 2009 to 2024

Country	Risk factors identified	Factors significantly associated	Author (year)
Ethiopia	Sex, age, residence, exposure to animals	History of animal contact	Abay et al. (2024) [27]
Ethiopia	Sex, age, residence, exposure to animals	History of animal contact	Behailu et al. (2022) [28]
Ethiopia	Residence	-	Belina et al. (2023) [29]
Uganda	Age	-	Mshana et al. (2009) [30]
Tanzania	Sex, age	-	Chercos et al. (2024) [31]
Zambia	Sex	-	Chiyangi et al. (2017) [32]
Tanzania	Sex, age, exposure to animals	Significantly higher in male	Chuma et al. (2016) [33]
Tanzania	Sex, age, exposure to animals	Significantly higher in children aged ≥ 24 months	Deogratias et al. (2014) [34]
Ethiopia	Sex, age, residence, exposure to animals	History of animal contact	Lengerh et al. (2013) [35]
Ethiopia	Age, sex	-	Getamesay et al. (2014) [36]
Ethiopia	Age, sex	Significantly higher in children aged < 1 year	Mulu et al. (2024) [37]
Ethiopia	Residence, exposure to animals	History of animal contact	Nigusu et al. (2022) [38]
Ethiopia	Age, sex, exposure to animals	History of animal contact	Tafa et al. (2014) [39]
Ethiopia	Age, sex, residence, exposure to animals	Residence; history of animal contact	Worku et al. (2024) [40]
Kenya	Age, sex	-	Zachariah et al. (2021) [41]
Kenya	Age, sex, residence, exposure to animals	Sex, residence, history of animal contact	Ouko et al. (2021) [42]
Malawi	Age	-	Mason et al. (2013) [43]
Kenya	Animal exposure	Animal contact	Conan et al. (2017) [44]
Kenya	Residence	-	Beatty et al. (2009) [46]
Tanzania	Age	-	Hugho et al. (2023) [55]
Kenya	Animal exposure	Animal contact	Kiarie et al. (2023) [16]
Ethiopia	Sex, animal exposure	-	Chen et al. (2021) [15]

**Table 3:** The reported status of thermophilic *Campylobacter* species among the included studies

Country	Overall prevalence	Prevalence in % (relative proportion) of:				Author (year)
		C. jejuni	C. coli	C. lari	C. upsaliensis	
Ethiopia	6.5%*	(-)	(-)	(-)	(-)	Abay et al. (2024) [27]
Ethiopia	6.8%	(-)	(-)	(-)	(-)	Behailu et al. (2022) [28]
Ethiopia	8.4%	(-)	(-)	(-)	(-)	Belina et al. (2023) [29]
Uganda	9.3%	7.5 (80.9%)	0.4 (4.5%)	0.9 (9.5%)	(-)	Mshana et al. (2009) [30]
Tanzania	10.9%	10.9 (100%)	(-)	(-)	(-)	Chercos et al. (2024) [31]
Zambia	1.1%	1.1 (100%)	(-)	(-)	(-)	Chiyangi et al. (2017) [32]
Tanzania	19.0%	14.9 (78.4%)	3.7% (19.6%)	(-)	(-)	Chuma et al. (2016) [33]
Tanzania	9.7%	(-)	(-)	(-)	(-)	Deogratias et al. (2014) [34]
Ethiopia	15.7%**	(-)	(-)	(-)	(-)	Lengerh et al. (2013) [35]
Ethiopia	12.7%	(-)	(-)	(-)	(-)	Getamesay et al. (2014) [36]
Ethiopia	19.5%	19 (96.6%)	1 (3.4%)	(-)	(-)	Mulu et al. (2024) [37]
Ethiopia	8.9%***	(-)	(-)	(-)	(-)	Nigusu et al. (2022) [38]
Ethiopia	16.7%	11.9 (71.1%)	3.5 (21.1%)	1.3 (7.9%)	(-)	Tafa et al. (2014) [39]
Ethiopia	7.0%	5.1 (73.3%)	1.9 (26.7%)	(-)	(-)	Worku et al. (2024) [40]
Kenya	12.9%	12.9 (100%)	(-)	(-)	(-)	Zachariah et al. (2021) [41]
Kenya	12.1%	10–12 (89.2%)	0.5–2 (10.8%)	(-)	(-)	Ouko et al. (2021) [42]
Malawi	21.4%	18.2 (85%)	3.2 (15%)	(-)	(-)	Mason et al. (2013) [43]
Kenya	19.2%	12.3 (64.3%)	6.8 (35.7%)	(-)	(-)	Conan et al. (2017) [44]
Tanzania	3.3%	3.3% (100%)	(-)	(-)	(-)	Oketcho et al. (2012) [45]
Kenya	4.9%	4.1.(82.5%)	0.9 (17.5%)	(-)	(-)	O’Reilly et al. (2012) [47]
Tanzania	2.4%***	(-)	(-)	(-)	(-)	Gosselin et al. (2017) [48]
Tanzania	4.7%	(-)	(-)	(-)	(-)	Mushi et al. (2014) [50]
Zambia	28.7%	(-)	(-)	(-)	(-)	Chisenga et al. (2018) [54]
Tanzania	3.4%	3.4% (100%)	(-)	(-)	(-)	Hugho et al. (2023) [55]
Madagascar	9.7%	7.3 (75.6%)	2.4 (24.4%)	(-)	(-)	Randremanana et al. (2012) [56]
Tanzania	39.1%***	(-)	(-)	(-)	(-)	Platts-Mills et al. (2014) [57]
Ethiopia	15%***	(-)	(-)	(-)	(-)	Terefe et al. (2020) [59]
Ethiopia	48.6%	(-)	(-)	(-)	(-)	Budge et al. (2020) [62]

(-) indicates species not specifically reported; * C. jejuni/C. coli accounts for 85.7% of cases; ** C. jejuni/C. coli accounts for 90.9% of cases; *** C. jejuni/C. coli accounts for 100% of all isolated

### Overall prevalence of *Campylobacter* infections in under-five children

**Fig. 2:**
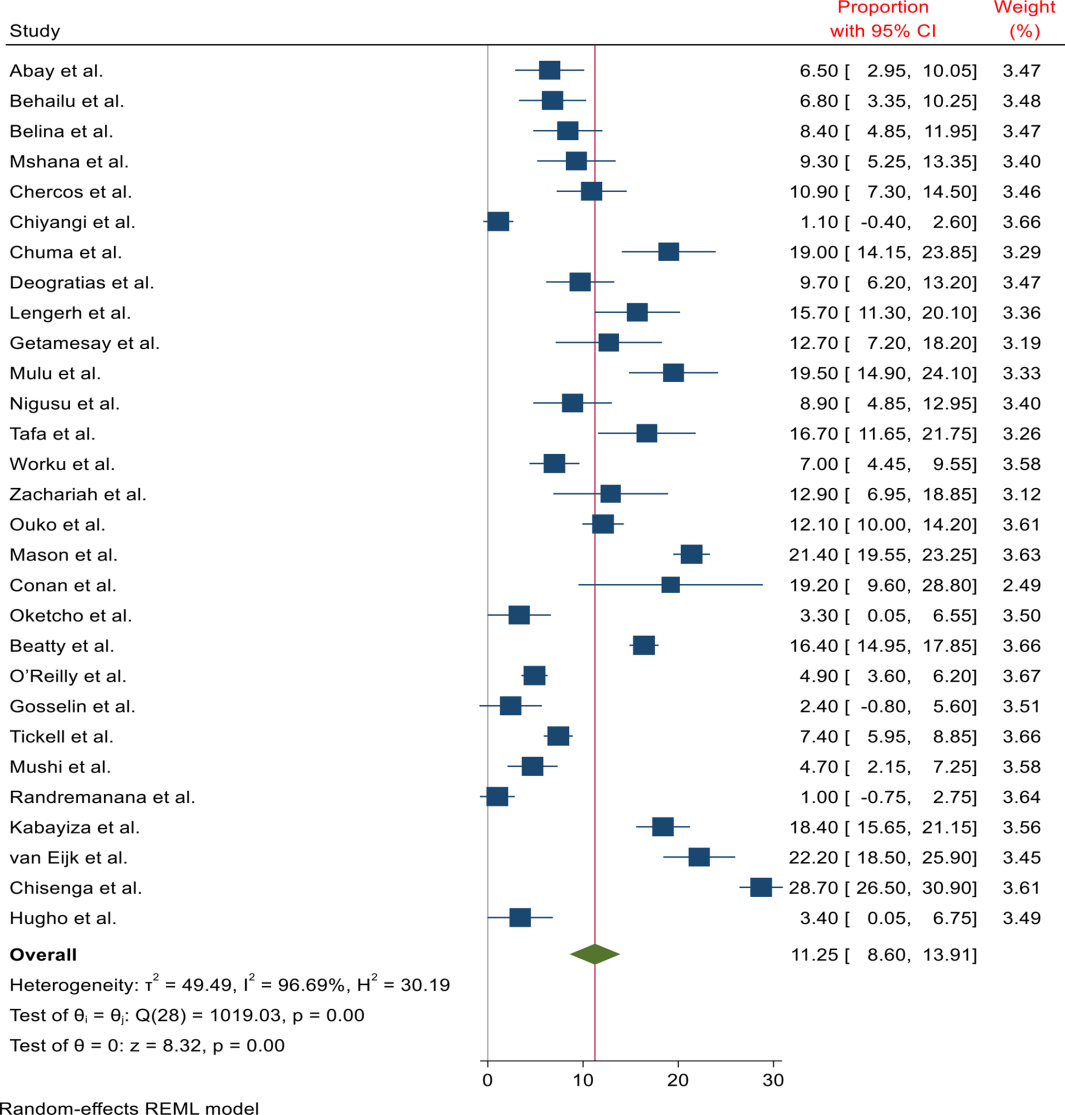
Forest plot of the pooled prevalence of *Campylobacter* occurrence in UFC in East Africa

**Fig. 3:**
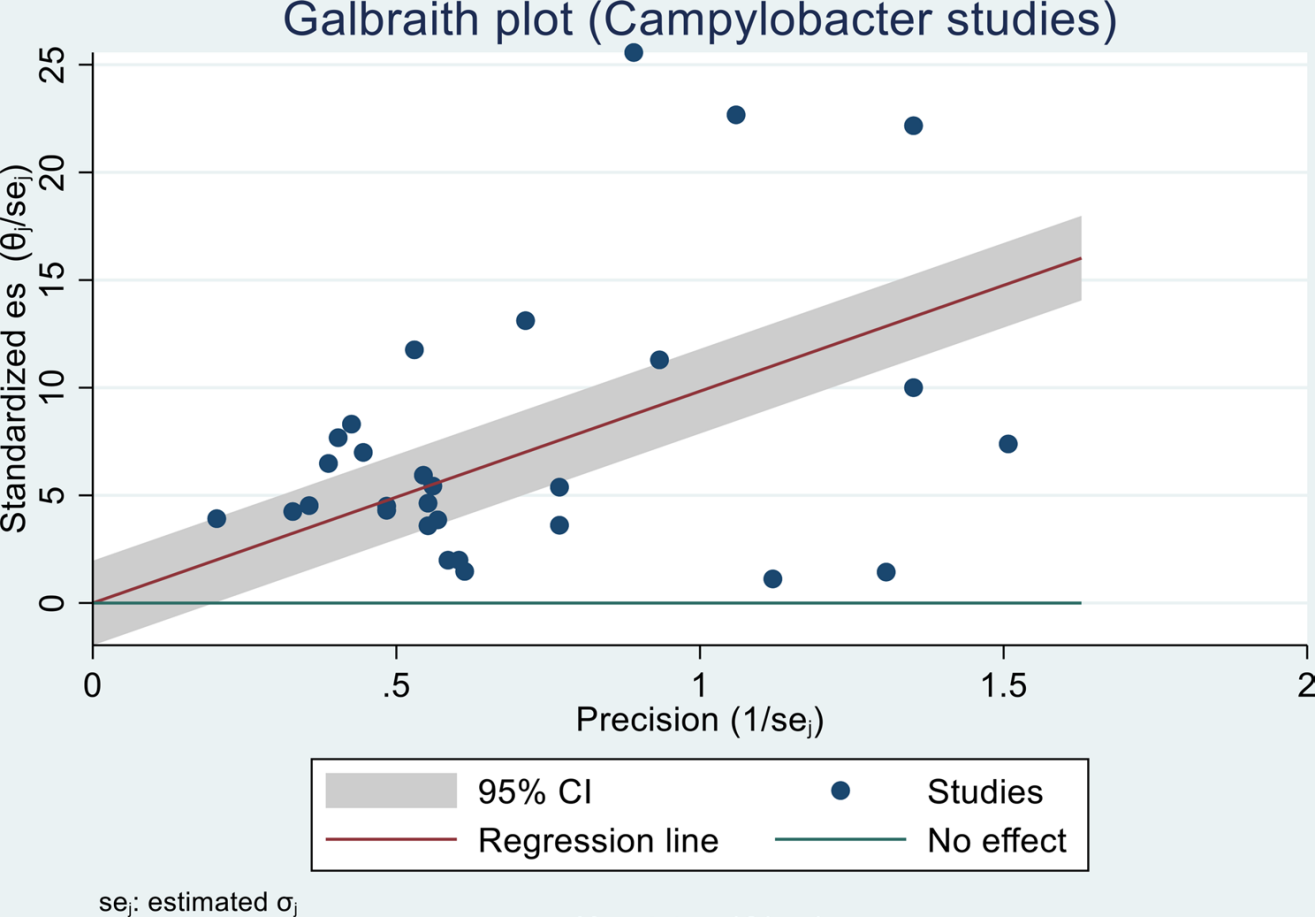
Galbraith plot of the pooled prevalence of *Campylobacter* occurrence in under-five children in East Africa

The overall pooled *Campylobacter* prevalence was analyzed by assuming a random effect model (Fig. 2). Due to the high degree of heterogeneity (I^2^ = 97%), an attempt was made based on subgroup analysis for pooled estimates. Moreover, the Galbraith plot showed that most studies were not within the prediction margin (Fig. 3). There was also publication bias as shown by the irregular patterns in the funnel plot (Figure Supplementary 1). The literature indicates that a high level of heterogeneity is inevitable when studies are particularly subjected to methodological variability in different population settings. However, this cannot prevent the meta-analysis of findings, as subgroup and meta-regression analyses are designed to address the high level of heterogeneity [20, 63].

#### Sub-group analysis

Method of detection (culture vs molecular), *Campylobacter* thermotolerance, geographic location (Horn of Africa vs others), elevation (1500 m.a.s.l. as a cut-off point), and year of publication (past five years as a cut-off point) were considered for the sub-group analysis. The study period was not considered, as two studies didn’t mention the year of study. Despite the subgroup analysis, the between-study heterogeneity remained higher (Figure Supplementary 2).

#### Meta-regression

Meta-regression was conducted to show the source of heterogeneity based on study size (numerical variable) and detection method (categorical variable) because more sensitive methods are used inconsistently across studies. So, the covariate study size explains roughly 18% of the between-study variance, while it was 15% for the detection method (Figure Supplementary 3).

Overall, the highest prevalence was reported from Ethiopia [61] at a proportion of 63.8%, while the lowest prevalence was reported from Zambia [32] and Madagascar [51] at a proportion of 1.0% (Table 1).

### Risk factors for thermophilic *Campylobacter* infections

From the included studies, data presentation based on sex, age, residence, and animal exposure was reported from Ethiopia, Kenya, Malawi, Tanzania, Uganda, and Zambia (Table 2).

Table 2. Summary of risk factors (sociodemographic and animal exposure) associated with UFC *Campylobacter* outcomes in East Africa from 2009 to 2024.

#### Children’s sex

**Fig. 4:**
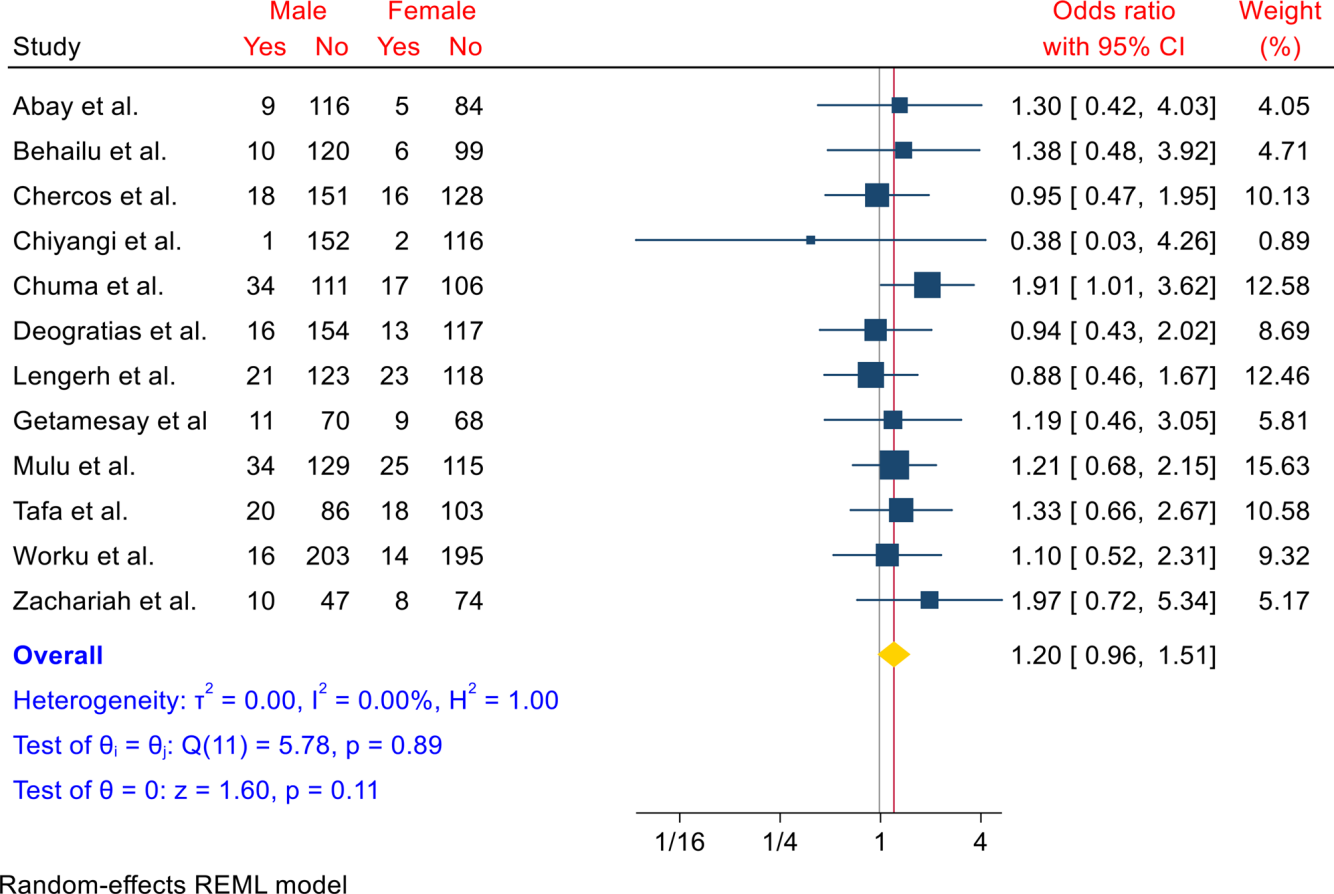
The pooled odds ratio of the association of sex with the prevalence of UFC thermophilic campylobacteriosis occurrence in East Africa

Twelve (12) studies were used to draw the pooled estimate of an association between sex and thermophilic campylobacteriosis (Fig. 4). Thus, males were 1.2 times (95% CI = 0.9–1.5) more likely to be positive for thermophilic *Campylobacter,* but the variation was statistically insignificant (*p* > 0.05).

#### Children’s age

Considering 12 and 24 months as a cut-off point, *Campylobacter* prevalence showed a high level of heterogeneity ( > 50%). Thus, subgroup analysis was conducted based on year of publication, geographic location, elevation, detection method, and *Campylobacter* spp. However, only the heterogeneity of 12-month age categorization was explained by the subgroup analysis of elevation, publication year, and geographic location. Thus, a pooled estimate was not determined for the age cut-off point as 24 months (Figure Supplementary 4).

##### Subgroup analysis of age

**Fig. 5:**
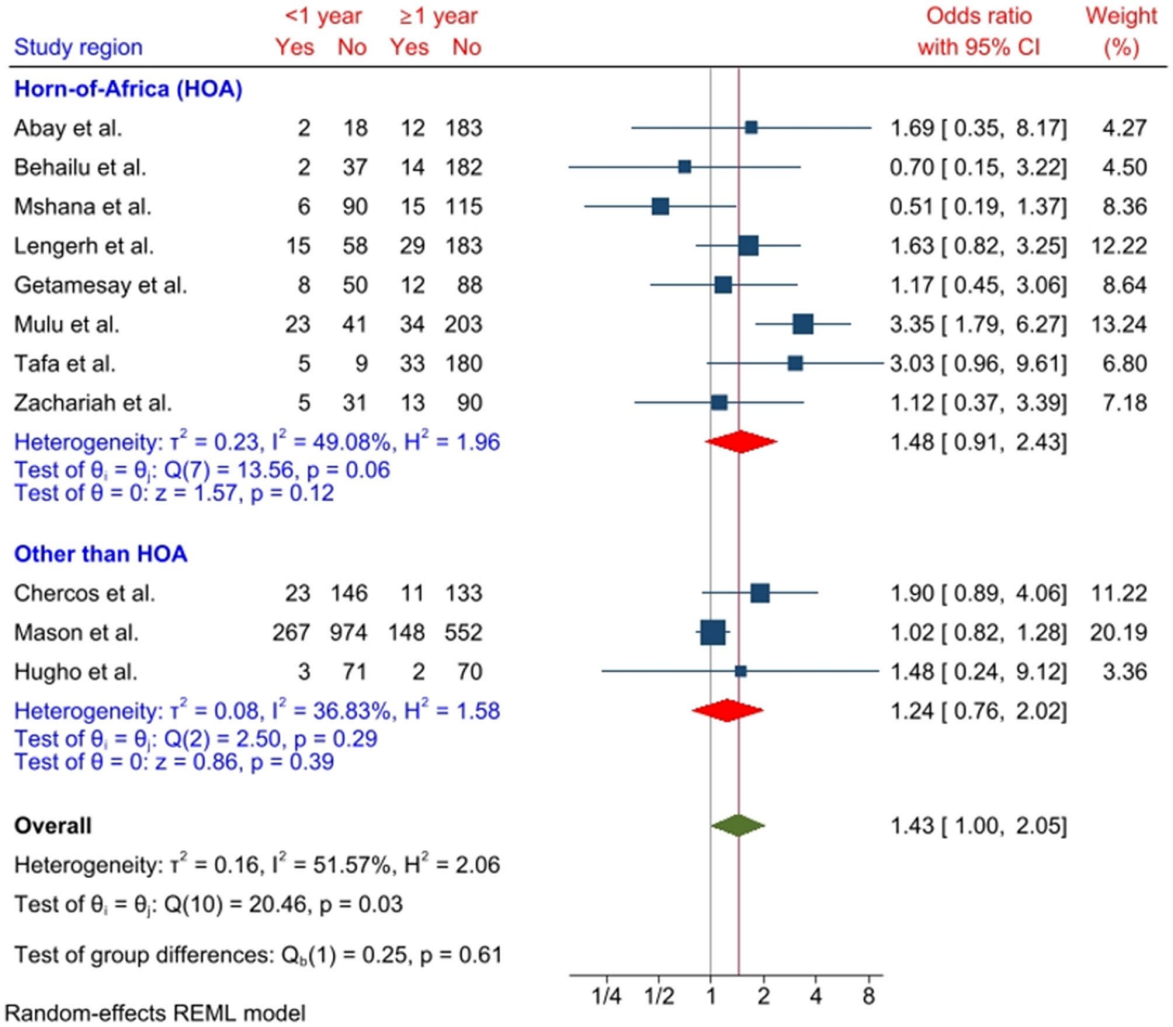
The pooled odds ratio of the association of age with the prevalence of thermophilic *Campylobacter* occurrence in under-five children in East Africa

**Fig. 6:**
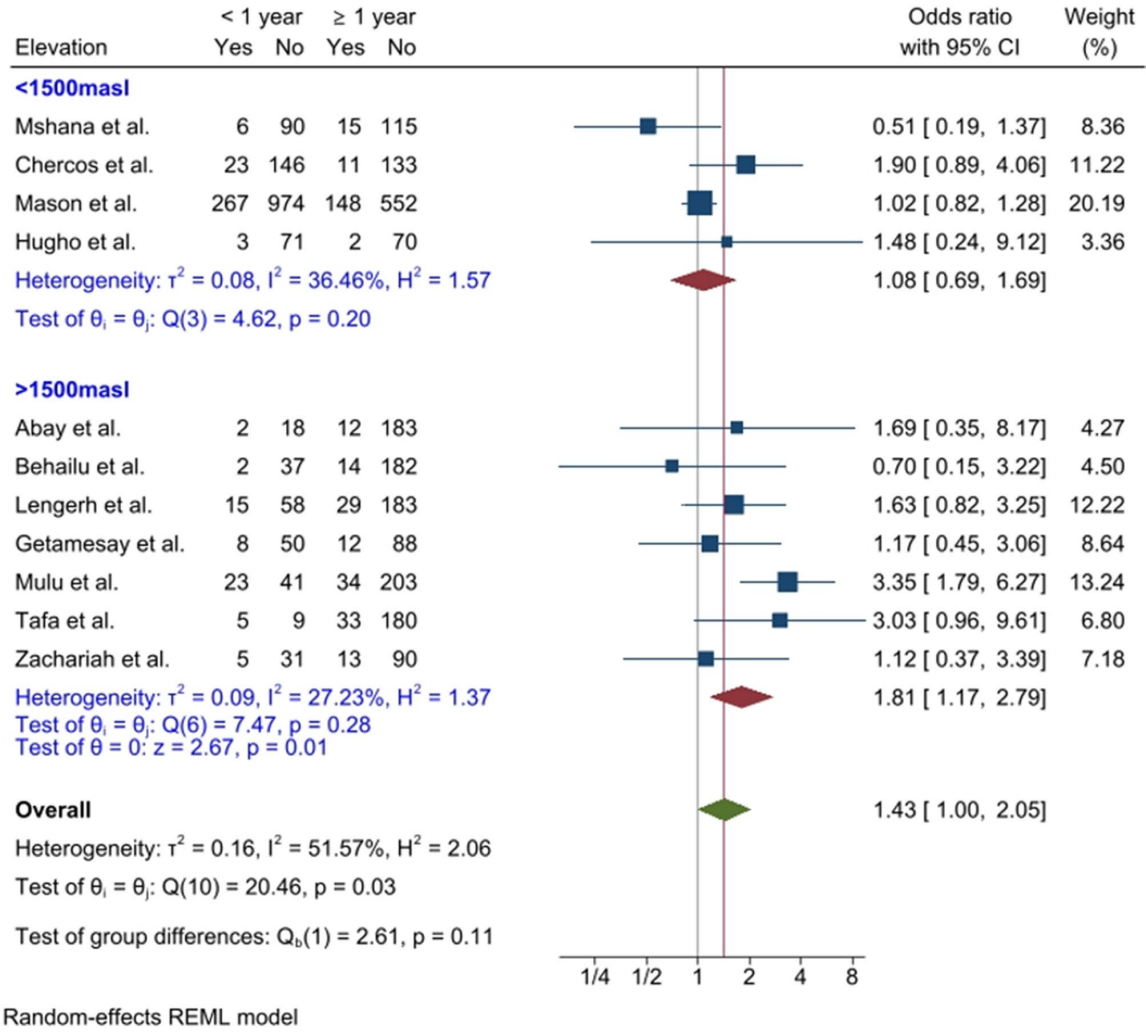
The pooled odds ratio of the association of elevation (m.a.s.l.) with prevalence of thermophilic *Campylobacter* occurrence in under-five children in East Africa

**Fig. 7:**
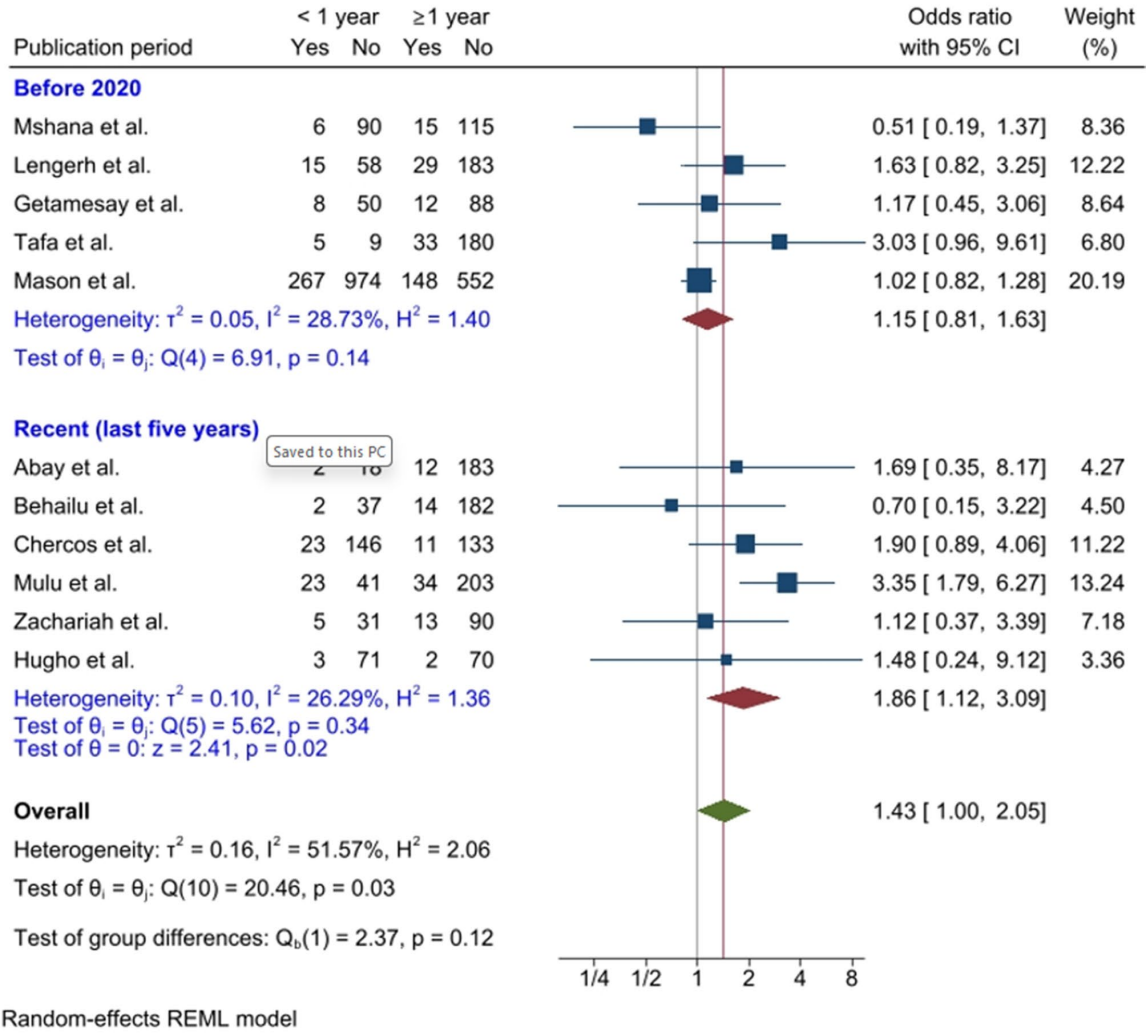
The pooled odds ratio of the association of publication period with prevalence of thermophilic *Campylobacter* occurrence in under-five children in East Africa

Based on the subgroup analysis, children in the Greater HoA and aged < 1 year had 1.5 times the odds (95% CI = 0.9–2.4) of being positive for thermophilic campylobacteriosis, but the association was insignificant (*p* = 0.12) (Fig. 5). Based on an average elevation of the study areas found at > 1500 m.a.s.l., the younger age category had 1.8 times (95% CI = 1.2–2.8; *p* = 0.01) the odds of being positive (Fig. 6). Concerning the period of publication, among studies published in the last five years (2020–2024), the younger children ( < 1 year) had 1.9 times (95% CI = 1.1–3.1; *p* = 0.02) the odds of being positive (Fig. 7).

##### Meta-regression analysis of age

Concerning age categorization with 24 months as a cut-off point, the covariate geographic location explains 67% of the between-study variance. The regression coefficient is −0.8 (*p* < 0.05), in that being in countries other than HoA corresponds to a decrease of ~0.8 units (*p* < 0.05) in log risk. Thus, we cannot proceed with meta-analysis.

Out of the 10 studies reported based on age classes < 24 months and older (Table 2), the majority (*n* = 8; 80%) reported higher prevalence in the younger age category from Ethiopia [27, 36–39], Kenya [41, 42], and Tanzania [31]. In contrast, Deogratias et al. [34] reported a significantly higher prevalence (*p* < 0.05) in the older age category (≥2 years) from Tanzania, in that children aged 24 months and above were 2.9 times more likely to be positive for campylobacteriosis when compared with the younger age group (≤24 months).

#### Association of animal exposure with campylobacteriosis

The pooled estimate of OR from data on animal exposure (Figure Supplementary 5) showed a high degree of heterogeneity ( > 50%), which couldn’t be explained by subgroup analysis based on geographic location, elevation, study period/year of publication, and *Campylobacter* spp. From the 12 studies documenting the association of animal exposure with campylobacteriosis (Table 2), nine (75%) showed a significantly higher campylobacteriosis within UFC exposed to animals than those not exposed. Thus, from Ethiopia, the reported OR ranges roughly from 4 to 11 [27, 28, 35, 38–40], while the reported OR ranges roughly from 2 to 6 in Kenya [16, 42, 44].

Some studies showed that age structure and type of animal exposure were linked with children’s campylobacteriosis. For instance, only children aged 2–5 years showed a significant association with exposure to domestic animals (OR = 5.7; 95% CI = 1.2–10.0) compared to the comparator group [42]. From Ethiopia, it was reported that children in contact with a cat had 11 times the chance of being positive for *Campylobacter* [28], while contact with other domestic animals didn’t show a significant association with children’s campylobacteriosis. Likewise, Lengerh et al. [35] reported that the likelihood of being positive for *Campylobacter* was 6 (95% CI = 2.3–11.7) and 3 (95% CI = 1.1–7.9) times higher in children exposed to cats/dogs and hens/pigeons, respectively, than in comparator groups, but failed to show a significant association (OR = 1.4: 95% CI = 0.5–5.4) when exposed to other animals.

#### Association of poultry exposure with campylobacteriosis

**Fig. 8:**
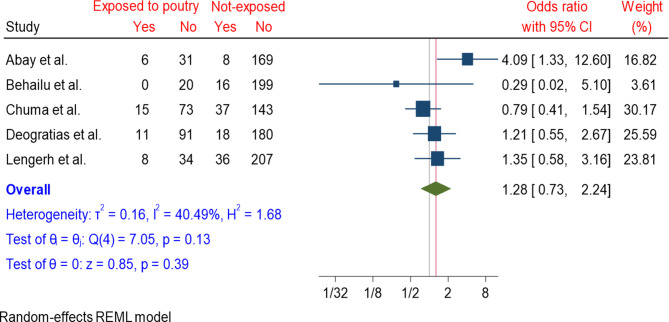
The pooled odds ratio of the association of poultry exposure with the prevalence of thermophilic *Campylobacter* occurrence in under-five children in East Africa

The pooled OR based on exposure to poultry was 1.3 (OR = 1.3; 95% CI = 0.7 - 2.2; *p* = 0.39), showing that the risk of thermophilic *Campylobacter* in children exposed to poultry is on average 30% higher compared to those not exposed (Fig. 8).

#### Association of residence exposure with campylobacteriosis

Six (6) studies were used to assess the pooled OR of association between residence and thermophilic campylobacteriosis. The heterogeneity test (Figure Supplementary 6) showed a high degree of between-study variability ( > 50.0%), and the between-studies variability couldn’t be explained by subgroup analysis based on reported *Campylobacter* spp.

Out of the eight studies included in this review, 63% (*n* = 5) reported a relatively higher prevalence in rural settings in Ethiopia [27, 35, 38, 40] and Kenya [42]. From these, Worku et al. [40] reported a significantly higher risk (OR = 5) in the rural area of Ethiopia. Likewise, children in urban areas of Kenya were 0.5 times (OR = 0.5; 95%CI = 0.2–0.8) less likely to be positive for thermophilic *Campylobacter* than those living in rural/village areas [42].

### Prevalence of *Campylobacter* species

Overall, 15, 10, and two studies provide a separate report on the status of *C. jejuni*, *C. coli*, and *C. lari*, respectively (Table 3). *C. lari* was only reported from Uganda (0.9%) by Mshana et al. [30] and Ethiopia (1.3%) by Tafa et al. [39]. Thus, pooled estimate analysis was only performed for *C. jejuni*, *C. coli,* and *C. jejuni/coli.* No study showed a separate report on *C. upseliansis*.

Table 3. The reported status of thermophilic *Campylobacter* species among the included studies.

#### The prevalence of *Campylobacter jejuni* among the included studies

In the meta-analysis, fourteen (14) reported separately on *C. jejuni*, but the heterogeneity test (Figure Supplementary 7) showed a high degree of between-studies variability ( > 50.0%). The variability cannot be handled by subgroup and meta-regression. The prevalence of *C. jejuni ranges* from 5.1–19.0% in Ethiopia [37, 39, 40], 4.1–12.9% in Kenya [41, 42, 44, 47], 3.3–14.9% in Tanzania [31, 33, 45, 55], 18.2% in Malawi [43], 7.5% in Uganda [30], 1.1% in Zambia [32], and 7.3% in Madagascar [56].

Overall, *C. jejuni* was the dominant isolate. Thus, some studies reported *C. jejuni* accounted for 80–96% of total isolates [30, 37, 42, 43, 47]. Studies from Tanzania [31, 45, 55], Zambia [32]), and Kenya [41] showed that *C. jejuni* was the sole species (100%) isolated from diarrheic children.

#### The prevalence of *Campylobacter coli* among the included studies

Nine (9) studies reported separately on *C. coli* but showed a high degree of heterogeneity ( > 50.0%). The variability was explained by geographical location, but a limited number of studies (only two) yielded an acceptable effect estimate, thus not considered for interpretation (Figure Supplementary 8). Additionally, there is a substantial level of small study effect from Egger’s test (beta1 = 1.9; *p* = 0.033).

#### The prevalence of *Campylobacter jejuni*/*coli*

Twelve (12) studies reporting on *C. jejuni/coli* were considered for meta-analysis. However, the heterogeneity test (Figure Supplementary 9) showed a high degree of between-studies variability ( > 50.0%), which cannot be handled by subgroup and meta-regression analysis. Thus, some studies showed that (Table 3) 100% of *Campylobacter* isolates from diarrheic cases were attributed to *C. jejuni/coli* in Ethiopia [27, 38] and Tanzania [48, 57].

### Risk of bias

The majority (~71%) of the included studies had a moderate risk of bias, while the remaining five (29%) studies [28, 29, 33, 38, 42] had a low risk of bias. In all the articles with moderate risk of bias, “unclear” was the most common response. The majority (10/17) didn’t rule out any impact from a concurrent/unintended intervention on the outcome of interest. All the studies included in the systematic review lack information about the blinding of the outcome assessors to the intervention or exposure status of participants. Also, valid and reliable measures of confounding variables were the limitations in all studies (Table Supplementary 5).

## Discussion

In UFC, death due to *Campylobacter* gastroenteritis varied by geographic region and income status, with high mortality in South Asia, Sub-Saharan Africa, Latin America/Caribbean, and low−middle sociodemographic index (SDI), but with a low mortality record in high-income countries [3]. Low-income countries have limited access to safe water, poor food handling facilities, and inadequate hygiene and sanitation practices [64, 65]. Also, the low-middle SDI is associated with an increasing burden of malnutrition [62, 66] and drug-resistant strains [67, 68], which contribute to Campylobacter complications.

In this systematic review, the prevalence range of 1.1–28.7% in health care settings is within the range of 10–40% reported from countries with low-resource settings [64]. Moreover, it is like the 12.3% reported in Georgia [69]; 10.1% India [70]; 21.5% in Lebanon [14]; and 6.8% in Egypt [13]. Likewise, the 5–63% in a community setting in East Africa is in line with the proportion of 50–70% reported from Bangladesh and Tanzania [64].

Detection methods play a critical role in shaping the magnitude of campylobacteriosis reporting. PCR is sensitive and can detect viable but non-cultivable organisms. However, they can also detect dead bacteria’s genetic material, which cannot tell the current status but can show recent infections [71]. Culture-based detection of isolates was less sensitive (50%) than PCR (100%), but both achieved 100% specificity [72]. Moreover, PCR tests for direct detection of *Campylobacter* from stool have been widely used in clinical settings and have high sensitivities (100%) and specificities (≥98%), with a detection limit of 66 CFU/mL [73].

Campylobacteriosis prevalence has also been related to season/climate, which is attributed to ambient temperature [67]. A lag period of 10–14 weeks before infection showed 1.3 odds of an increase per 1 °C increase in temperature [74]. At a lag period of two weeks, an increased incidence of 0.13 per 1 °C temperature increase was reported in the window of value 5.1 to 12.2 °C [75]. In temperate areas, the expected cases of campylobacteriosis increase when the average temperature two weeks before diagnosis exceeds 11 °C, and temperature contributed to a 33.3% increase in expected *Campylobacter* cases [76]. From a meta-analysis finding, a 1 °C increase in temperature increases the risk of campylobacteriosis by 5% [77].

Other determinants of campylobacteriosis include sanitation, proximity to animals, food consumption habits, and immune status [78]. Thus, factors significantly associated with increased risk include drinking undisinfected water, eating poultry bought raw, occupational exposure to animals, eating undercooked pork, and sharing a meal at a community cookout [79]. The heightened vulnerability of the immunocompromised individuals is evident by the observation of exceptionally high infection rates (50.7%) among HIV-positive patients in some settings [80]. The pooled estimate showed that males were 1.2 times (95% CI = 0.9–1.5; *p* > 0.05) more likely to be positive for thermophilic *Campylobacter*. This partly agrees with the report from India [81]; New Zealand [82]; Lebanon [14]; Taiwan [12]; and the United States of America [83], which reported significantly more notifications for males than females. On the contrary, Samie et al. [84] from South Africa reported significantly higher prevalence in females but only with the *C. jejuni* isolates. Moreover, a birth cohort study at eight sites in South America, sub-Saharan Africa, and Asia [Haque11]; Georgia [67]; Ethiopia [15]; and India [68] reported relatively higher prevalence in female children. Generally, the observed differences are inconsistent across different countries and time periods, and there is no gender-based biological description of campylobacteriosis. However, a recent meta-analysis study has shown that the predominance of campylobacteriosis in males starts in infancy, suggesting physiological or genetic differences apart from behavioral factors to play a role in the pathogenesis of campylobacteriosis [85].

In countries of the Greater HoA, the observed higher risk of thermophilic campylobacteriosis in children aged < 1 year (OR = 1.5; 95% CI = 0.9–2.4) agrees with the report that in infants ( < 1 year of age), breastfeeding activities might influence gut colonization with beneficial and harmful pathogens such as thermophilic *Campylobacter* species [86]. On the contrary, a higher prevalence was reported in children aged ≥ 1 year from India [81]. Generally, it was indicated that the prevalence of *C. jejuni/C. coli* and other *Campylobacter* spp. increased sharply in the first year of age. However, the association between children’s age and the prevalence of *Campylobacter* is best explained by the variation in acquisition rate, in which a high acquisition rate was reported from 11 to 17 months of age [87]. In the systematic review, the reported higher prevalence in children less than < 2 years from Ethiopia [27, 36–39]; Kenya [41, 42] and Tanzania [31] is supported by the report from northern India, in that the prevalence of *Campylobacter* diarrhea was higher in children up to 24 months compared to older children, but it was statistically insignificant [68]. In developing countries, *Campylobacter* infections in children under the age of 2 years are especially frequent, sometimes resulting in death [5, 78].

We used publication year as a proxy for the study period due to incomplete reporting. To avoid a mislead in trend interpretations due to the use of publication year, we conducted an analysis when the study period was available. Thus, it showed minimal changes in effect size (Figure Supplementary 10) but significant shifts in statistical significance. In the first subgroup (recent category), the odds ratio and its corresponding p-value shifted from 1.86 (*p* = 0.02) to 1.87 (*p* = 0.23), and in the second subgroup, it changed from 1.15 (*p* = 0.14) to 1.25 (*p* = 0.22). This indicates that while the effect sizes were stable, the loss of significance was likely due to smaller sample sizes, requiring many more included studies [88]. This suggests that reports based on the publication period should be interpreted with caution. Also, these findings underscore the need for complete reporting of study periods and confirm our decision to use publication year to enhance study inclusion and strengthen the results.

Most of the studies in the systematic review reported that campylobacteriosis was significantly associated with children’s history of contact with animals, such as those from Ethiopia [27, 28, 35, 38–40] and Kenya [16, 42, 44]. These findings coincide with the report from Lebanon [14]. In the current meta-analysis, the higher risk of thermophilic *Campylobacter* in children exposed to poultry coincides with earlier reports. Thus, ownership of pet chickens and pet puppies was significantly associated with illness of children aged 0–35 months in Australia [89]. Likewise, contact with poultry was a significant risk for *C. jejuni/C. coli* gastroenteritis in Germany [90], and infant campylobacteriosis was positively and significantly associated (*p* = 0.003) with keeping fowl in the home in Egypt [13]. In some areas, not all forms of animal exposure are detrimental. For instance, in Denmark, a significant association was recorded only when exposure status was based on contact with cattle. For UFC, the likelihood of acquiring the disease was 62.4 times higher (ranged 8.2–472.6) than those with no contact with animal feces [91]. From Egypt, El-Tras et al. [92] showed that household chicken campylobacteriosis was positively associated with children’s infection with *C. jejuni*. Moreover, Chen et al. [15] have documented that *Campylobacter* occurrence in children aged 12–16 months was higher in houses where chickens are kept inside the home at night. Within a household, the *Campylobacter* load in infant stool samples was positively correlated with *Campylobacter* loads in chicken and cattle feces [61]. From Peru, Oberhelman et al. [93] documented that similar strains of *Campylobacter* in chickens and humans were found in many family clusters, especially those with the largest number of bacterial isolates. Poultry, specifically chickens, have a metabolic temperature of 42 °C, leading to an optimal environment for *Campylobacter* growth. Additional characteristics, such as the sudden stops of growth below 30 °C, and the inability to survive under the ambient oxygen level, can limit but not eliminate their prevalence outside warm-blooded hosts in foods and/or food environments [94]. Thus, in developing countries, close contact with animals, including chickens, was found to be an important risk factor for acquiring the infection [95].

About residence, the relatively higher prevalence in rural settings by most of the studies parallels a report from Denmark, in that those who live in urban areas have a low likelihood of encountering campylobacteriosis [91]. The increased risk of individuals in rural areas is due to occupational exposure to animals and the consumption of private well water [96]. The spatial and temporal determinants of campylobacteriosis are reported to be complex. The increased risk of rural residence for young children is environmental exposure, such as direct contact with farm animals or their feces, swimming in lakes and rivers, and drinking untreated water [82, 83].

The prevalence of thermophilic species varies from country to country; however, most of the cases were attributed to C. *jejuni,* followed by *C. coli*, which is in line with earlier findings [86]. In addition, 81% of cases were due to *C. jejuni*, while the remaining 19% attributed to *C. coli* [67]. Borkakoty et al. [68] detected *C. jejuni* in 80.5% of cases, and Ibrahim et al. [14] reported *C. jejuni* (83.2%) and *C. coli* (13.9%). In developed nations such as the United Kingdom and the United States of America, *C. jejuni* typically accounts for 90% of cases, while *C. coli* accounts for the remaining ≏10% of cases [8].

The majority (60.5%) of the studies included in the current review had a moderate risk of bias. Agreeably, earlier prevalence studies reported that the majority (64%) had a moderate risk of bias [97]. On the contrary, Diriba et al. [18] report that almost all of the studies considered for systematic review and meta-analysis fall under low risk of bias. These disparities could be attributed to the application of different assessment criteria. Moreover, the limited number of studies (*n* = 8) considered for evaluation by Diriba et al. [18] could not be conclusive. The major sources for increased risk of bias include a lack of information about the blinding of the outcome assessors, the absence of implementing valid and reliable measures of confounding variables, and inadequate criteria to rule out the impact of concurrent intervention or an unintended exposure in the outcome measures. Agreeably, a study based on an assessment of published articles in prestigious occupational medicine and health journals showed that most studies published in didn’t describe any efforts to address potential sources of bias [98].

## Limitations of the systematic review and meta-analysis

The major limitation was the lack of sufficient studies for the systematic review. Another limitation was the lack of consideration of metrological data (temperature, rainfall, and humidity) to address the high level of study heterogeneity. Studies reporting *Campylobacter* prevalence in ranges (with no specific figure) are excluded from the meta-analysis. A publication period as a variable may lead to temporal trend distortion. This review did not attempt to synthesize the prevalence of thermophilic campylobacteriosis based on risk factors associated with children’s nutritional status, breastfeeding activity, and socioeconomic status of parents/caregivers, because the reviewed studies had different exposure status measurement parameters. Moreover, information about the season of diarrheal occurrences and climatic conditions of the study areas is not gathered.

## Confidence in the level of evidence

Considering the number of studies with adequate sample size, sufficient coverage of the identified sample size (good response rate), valid methods used for the identification of the bacteria (thermophilic *Campylobacter* spp.), and appropriateness of the statistical analysis, the systematic review and meta-analysis evidence can be classified as good and reliable. However, the pooled estimate for *C. coli* based on meta-analysis is poor in reliability, due to the significant effect of the small study size.

## Conclusion

There is a wide range of variations in *Campylobacter* prevalence in UFC in East Africa, which can be influenced by study size, study year, and method of detection. Although the association did not reach statistical significance (*p* > 0.05), our finding suggests a potential trend toward increased risk in male children. Despite the evidence presented about age being inconsistent, most studies reported a higher occurrence in younger ( < 24 months) than older age. Geographic location and elevation (m.a.s.l.) influence the occurrence of thermophilic *Campylobacter* spp in different age categories. Exposure to poultry is a potential risk for UFC campylobacteriosis, showing the importance of practicing avoiding/limiting contact with animals. Generally, substantial studies found an association between animal exposure and campylobacteriosis in diarrheic UFC. *Campylobacter jejuni* was the most reported species, often detected in 60–100% of diarrheic UFC. Future studies on campylobacteriosis in children should consider substantial evidence by recommended age group categorization, exposure to specific animal species, and exposure to different climatic conditions. In addition, it is important to consider a separate result presentation for both thermophilic and non-thermophilic *Campylobacter* species.

## Electronic supplementary material

Below is the link to the electronic supplementary material.

Supplementary material 1

Supplementary material 2

## Data Availability

Materials related to template data collection forms; data extracted from included studies; and articles used in the review can be provided upon requesting the authors.
