# Supplementary material for: Prevalence and determinants of Campylobacter infection in under-five children of East Africa: systematic review and meta-analysis
Source: BMC Infect Dis. 2026 Jan 14;26:307. doi: 10.1186/s12879-026-12538-w (PMC12888365; doi:10.1186/s12879-026-12538-w)
Supplement: Supplementary file 1 — Supplementary material 1 [file 12879_2026_12538_MOESM1_ESM.docx]

Figure Supplementary 1. Funnel plot of the pooled prevalence of *Campylobacter* occurrence in under-five children in East Africa.

Figure Supplementary 2A-2E. Forest plot of sub-group analysis for *Campylobacter* occurrence in under-five children of East African Region.

| Figure Supplementary 2A. |
| --- |
|  |

| Figure Supplementary 2B |
| --- |
|  |

| Figure Supplementary 2C |
| --- |
|  |

| Figure Supplementary 2D |
| --- |
|  |

| Figure Supplementary 2E. |
| --- |
|  |

Figure Supplementary 3A-3B. Meta-regression of the covariates study size and detection method for Campylobacter occurrence in under-five children of East African Region.

| Figure Supplementary 2A |
| --- |
|  |

| Figure Supplementary 3B. |
| --- |
|  |

| Figure Supplementary 4. The pooled odds ratio of the association of age (24 months as cut-off point) with prevalence of thermophilic *Campylobacter* occurrence in under-five children in the East African Region. |
| --- |
|  |

| Figure Supplementary 5. The pooled odds ratio of the association animal exposure with the prevalence of thermophilic *Campylobacter* occurrence in under-five children in the East African Region. |
| --- |
|  |

| Figure Supplementary 6. The pooled odds ratio of the association residence with the prevalence of thermophilic *Campylobacter* occurrence in under-five children in the East African Region. |
| --- |
|  |

| Figure Supplementary 7. The pooled prevalence of *Campylobacter* *jejuni* in under-five children in the East African Region. |
| --- |
|  |

| Figure Supplementary 8. The pooled prevalence of *Campylobacter* *coli* in under-five children in East Africa. |
| --- |
|  |

| Figure Supplementary 9. The pooled prevalence of *Campylobacter* *jejuni*/*coli* in under-five children in the East African Region. |
| --- |
|  |

| Figure Supplementary 10. The pooled odds ratio of the association of study period with prevalence of thermophilic *Campylobacter* occurrence in under-five children in East Africa. |
| --- |
| 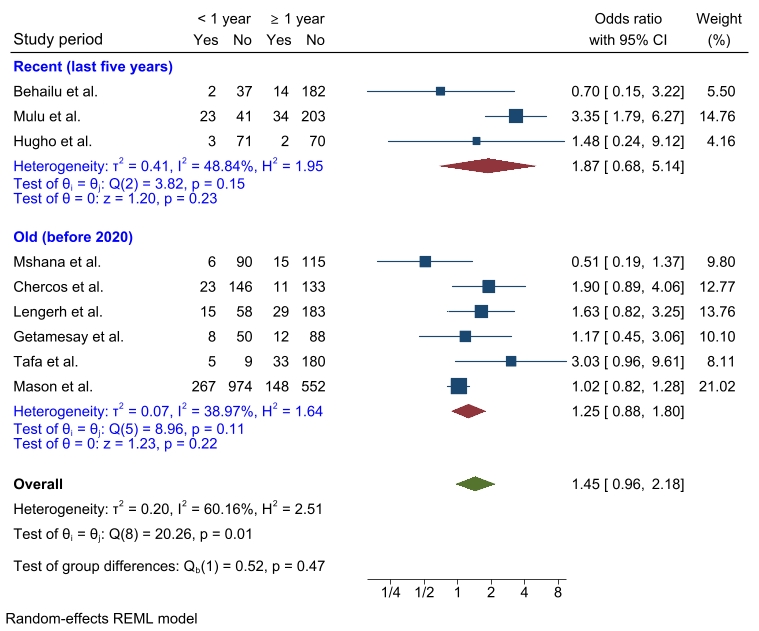 |
