# Supplementary material for: Prevalence and determinants of Campylobacter infection in under-five children of East Africa: systematic review and meta-analysis
Source: BMC Infect Dis. 2026 Jan 14;26:307. doi: 10.1186/s12879-026-12538-w (PMC12888365; doi:10.1186/s12879-026-12538-w)
Supplement: Supplementary file 2 — Supplementary material 2 [file 12879_2026_12538_MOESM2_ESM.docx]

Table Supplementary 1. The search queries are used for gathering relevant records from various databases.

| Name of the database | Features | Search query used |
| --- | --- | --- |
| PubMed/MEDLINE | - The use of Boolean terms such as AND, OR, NOT - Medical subject heading (MeSH) terms | ((((((((*Campylobacter*) OR (*Campylobacter jejuni*)) OR (*Campylobacter coli*)) OR (*Campylobacter lari*)) OR (*Campylobacter upsaliensis*)) OR (thermophilic *Campylobacter*)) AND (children)) OR (under-five children)) AND (Name of a country) |
| Research4life programmes | - The use of Boolean terms such as AND, OR - Subject terms | (*Campylobacter*) AND ((children) OR (under-five)) AND (name of a country) |
| Wiley Online Library | - The use of Boolean terms such as AND, OR, NOT - Wildcard searches such as *Campylobacter* *, child*, under* | "*Campylobacter* AND children AND the name of a country" |
| ScienceDirect | - The use of Boolean terms such as AND, OR, NOT - Punctuation is ignored in a phrase search - No need for wild cards | (*Campylobacter* OR name of the species) AND (children OR under-five) AND (name of a country) |

Table Supplementary 2. The JBI critical appraisal checklist used for studies reporting prevalence data (Munn *et al*., 2015)[20].

| Question # | Item |
| --- | --- |
|  | Was the sample frame appropriate to address the target population? |
|  | Were study participants sampled in an appropriate way? |
|  | Was the sample size adequate? |
|  | Were the study subjects and the setting described in detail? |
|  | Was the data analysis conducted with sufficient coverage of the identified sample? |
|  | Were valid methods used for the identification of the condition? |
|  | Was the condition measured in a standard, reliable way for all participants? |
|  | Was there appropriate statistical analysis? |
|  | Was the response rate adequate, and if not, was the low response rate managed appropriately? |

Table Supplementary 3. Assessing the Risk of Bias of Individual Studies in Systematic Reviews of Health Care Interventions (Viswanathan, 2012)[26].

| Item # | Description |
| --- | --- |
| 1 | Did the study apply inclusion/exclusion criteria uniformly to all comparison groups? |
| 2 | Does the design or analysis control account for important confounding and modifying variables through matching, stratification, multivariable analysis, or other approaches? |
| 3 | Did researchers rule out any impact from a concurrent intervention or an unintended exposure that might bias results? |
| 4 | If attrition (overall or differential nonresponse, dropout, loss to follow-up, or exclusion of participants) was a concern, were missing data handled appropriately (e.g., intention-to-treat analysis and imputation)? |
| 5 | Were the outcome assessors blinded to the intervention or exposure status of participants? |
| 6 | Were interventions/exposures assessed/defined using valid and reliable measures, implemented consistently across all study participants? |
| 7 | Were outcomes assessed/defined using valid and reliable measures, implemented consistently across all study participants? |
| 8 | Were confounding variables assessed using valid and reliable measures, implemented consistently across all study participants? |
| 9 | Were the potential outcomes prespecified by the researchers? Are all prespecified outcomes reported? |
| 10 | Did the study maintain fidelity to the intervention protocol? |
| 11 | In prospective studies, was the length of follow-up different between the groups, or in case-control studies, was the time period between the intervention/exposure and outcome the same for cases and controls? |
| 12 | Were cases and controls selected appropriately (e.g., appropriate diagnostic criteria or definitions, equal application of exclusion criteria to case and controls, sampling not influenced by exposure status)? |
| 13 | Were participants analyzed within the groups they were originally assigned to? |
| 14 | Did the strategy for recruiting participants into the study differ across study groups? |
| NB: Item # 1 is for both cross-sectional and cohort studies; *8 items (from # 2-9) are shared by all the observational studies*; Items # 10, 11 only applies to case-control and cohort; Items # 12 is only for case-control; Items # 13, 14 only applies to cohort. | |

Table Supplementary 4. The quality score of selected articles according to the JBI critical appraisal checklist developed for studies reporting prevalence data (Munn *et al*., 2015) [20].

| SN | Author, year | Question # | | | | | | | | | % Yes |
| --- | --- | --- | --- | --- | --- | --- | --- | --- | --- | --- | --- |
|  |  | 1 | 2 | 3 | 4 | 5 | 6 | 7 | 8 | 9 |  |
|  | Abay *et al*. (2024) | Y | Y | Y | Y | Y | Y | Y | Y | Y | 100 |
|  | Behailu *et al*. (2022) | Y | Y | Y | Y | Y | Y | Y | Y | Y | 100 |
|  | Belina *et al*. (2023) | Y | Y | Y | Y | Y | Y | Y | Y | Y | 100 |
|  | Mshana *et al*. (2009) | Y | Y | Y | Y | U | Y | Y | Y | Y | 88.9 |
|  | Chercos *et al*. (2024) | Y | Y | Y | Y | Y | Y | Y | Y | U | 88.9 |
|  | Chiyangi *et al*. (2017) | Y | U | Y | Y | Y | Y | Y | Y | U | 77.8 |
|  | Chuma *et al*. (2016) | Y | Y | Y | Y | Y | Y | Y | Y | Y | 100 |
|  | Deogratias *et al*. (2014) | Y | Y | Y | Y | Y | Y | Y | Y | Y | 100 |
|  | Lengerh *et al*. (2013) | Y | Y | Y | Y | Y | Y | Y | Y | U | 88.9 |
|  | Getamesay *et al*. (2014) | Y | Y | N | Y | Y | Y | Y | Y | U | 77.8 |
|  | Mulu *et al*. (2024) | Y | Y | Y | Y | Y | Y | Y | Y | U | 88.9 |
|  | Nigusu *et al*. (2022) | Y | Y | Y | Y | Y | Y | Y | Y | Y | 100 |
|  | Tafa *et al*. (2014) | Y | Y | Y | Y | Y | Y | Y | Y | Y | 100 |
|  | Worku *et al*. (2024) | Y | Y | Y | Y | Y | Y | Y | Y | Y | 100 |
|  | Zachariah *et al*. (2021) | Y | Y | Y | Y | Y | Y | Y | Y | Y | 100 |
|  | Ouko *et al*. (2021) | Y | U | Y | Y | Y | Y | Y | Y | U | 77.8 |
|  | Mason *et al*. (2013) | Y | U | Y | Y | Y | U | Y | Y | Y | 77.8 |
|  | Conan et al. (2017) | Y | Y | Y | Y | Y | Y | Y | Y | Y | 100 |
|  | Oketcho et al. (2012) | Y | Y | Y | Y | Y | Y | Y | Y | Y | 100 |
|  | Beatty et al. (2009) | Y | Y | Y | Y | Y | Y | Y | Y | Y | 100 |
|  | O’Reilly et al. (2012) | Y | Y | Y | Y | Y | Y | Y | Y | Y | 100 |
|  | Gosselin et al. (2017) | Y | N | Y | Y | Y | Y | Y | Y | Y | 88.9 |
|  | Tickell et al. (2017) | Y | Y | Y | Y | Y | Y | Y | Y | Y | 100 |
|  | Mushi et al. (2014) | U | Y | Y | U | Y | Y | Y | Y | Y | 77.8 |
|  | Randremanana et al. (2016 | Y | Y | Y | Y | Y | Y | Y | Y | Y | 100 |
|  | Kabayiza et al. (2014) | Y | Y | Y | U | Y | Y | Y | Y | Y | 88.9 |
|  | van Eijk et al. (2010) | Y | Y | U | Y | Y | Y | Y | Y | Y | 88.9 |
|  | Chisenga et al. (2018) | Y | Y | Y | Y | Y | Y | Y | Y | Y | 100 |
|  | Hugho et al. (2023) | Y | Y | Y | Y | Y | Y | Y | Y | Y | 100 |
|  | Randremanana et al. (2012) | Y | U | Y | Y | Y | Y | Y | Y | Y | 88.9 |
|  | Platts-Mills et al. (2014) | Y | U | N | Y | Y | Y | Y | Y | Y | 77.8 |
|  | McQuade et al. (2020) | Y | U | Y | Y | Y | Y | Y | Y | Y | 88.9 |
|  | Terefe et al. (2020) | Y | U | N | Y | Y | Y | Y | Y | Y | 77.8 |
|  | Kiarie et al. (2023) | Y | Y | Y | Y | U | Y | Y | Y | Y | 88.9 |
|  | Chen et al. (2021) | Y | Y | Y | Y | Y | Y | Y | Y | Y | 100 |
|  | Berendes et al. (2019) | Y | Y | N | Y | Y | Y | Y | Y | Y | 88.9 |
|  | Deblais et al. (2023) | Y | Y | Y | Y | Y | Y | Y | Y | Y | 88.9 |
|  | Budge et al. (2020) | Y | Y | N | Y | U | Y | Y | Y | Y | 77.8 |
| *Key: Y= Yes; N= No; U= Unclear.* | | | | | | | | | | | |

Table Supplementary 5. Risk of bias summary using the AHRQ tool for Risk of Bias of Individual Studies in Systematic Reviews of Health Care Interventions.

|  | **Q1** | **Q2** | **Q3** | **Q4** | **Q5** | **Q6** | **Q7** | **Q8** | **Q9** | **Q10** | **Q11** | **Q 12** | **Q 13** | **Q 14** | **Bias %** |
| --- | --- | --- | --- | --- | --- | --- | --- | --- | --- | --- | --- | --- | --- | --- | --- |
| Abay *et al*., 2024 | Y | Y | U | Y | U | Y | Y | N | Y | NA | NA | NA | NA | NA | 66.7 |
| Behailu *et al*., 2022 | Y | Y | Y | Y | U | Y | Y | N | Y | NA | NA | NA | NA | NA | 77.8 |
| Belina *et al*., 2023 | Y | Y | Y | Y | U | Y | Y | N | Y | NA | NA | NA | NA | NA | 77.8 |
| Mshana *et al*., 2009 | Y | Y | U | Y | U | Y | Y | N | Y | NA | NA | NA | NA | NA | 66.7 |
| Chercos *et al*., 2024 | NA | Y | U | U | U | Y | Y | N | Y | U | Y | Y | NA | NA | 63.6 |
| Chiyangi *et al*., 2017 | Y | Y | U | U | U | Y | Y | N | Y | NA | NA | NA | NA | NA | 55.5 |
| Chuma *et al*., 2016 | Y | Y | Y | Y | U | Y | Y | N | Y | NA | NA | NA | NA | NA | 77.8 |
| Deogratias *et al*., 2014 | Y | Y | U | Y | U | Y | Y | N | Y | NA | NA | NA | NA | NA | 66.7 |
| Lengerh *et al*., 2013 | Y | Y | Y | U | U | Y | Y | N | Y | NA | NA | NA | NA | NA | 66.7 |
| Getamesay *et al*., 2014 | Y | Y | Y | U | U | Y | Y | N | Y | NA | NA | NA | NA | NA | 66.7 |
| Mulu *et al*., 2014 | Y | Y | U | U | U | Y | Y | N | Y | NA | NA | NA | NA | NA | 55.6 |
| Nigusu *et al*., 2022 | Y | Y | Y | Y | U | Y | Y | N | Y | NA | NA | NA | NA | NA | 77.8 |
| Tafa *et al*., 2014 | Y | Y | U | Y | U | Y | Y | N | Y | NA | NA | NA | NA | NA | 66.7 |
| Worku *et al*., 2024 | Y | Y | U | Y | U | Y | Y | N | Y | NA | NA | NA | NA | NA | 66.7 |
| Zachariah *et al*., 2021 | Y | Y | U | Y | U | Y | Y | N | Y | NA | NA | NA | NA | NA | 66.7 |
| Ouko *et al*. (2021) | Y | Y | Y | Y | U | Y | Y | N | Y | NA | NA | NA | NA | NA | 77.8 |
| Mason *et al*. (2013) | Y | Y | U | U | U | Y | Y | N | Y | NA | NA | NA | NA | NA | 55.6 |
| Conan et al. (2017) | NA | Y | U | Y | U | Y | Y | Y | Y | Y | Y | Y | NA | NA | 81.8 |
| Oketcho et al. (2012) | Y | Y | U | Y | U | Y | Y | Y | Y | NA | NA | NA | NA | NA | 77.8 |
| Beatty et al. (2009) | Y | Y | U | Y | U | Y | Y | N | Y | NA | NA | NA | NA | NA | 66.7 |
| O’Reilly et al. (2012) | Y | Y | U | Y | U | Y | Y | Y | Y | NA | NA | NA | NA | NA | 77.8 |
| Gosselin et al. (2017) | Y | Y | U | U | U | Y | Y | N | Y | U | Y | NA | Y | Y | 61.5 |
| Tickell et al. (2017) | Y | Y | U | Y | U | Y | Y | Y | Y | NA | NA | NA | NA | NA | 77.8 |
| Mushi et al. (2014) | Y | N | U | Y | U | Y | Y | N | Y | NA | NA | NA | NA | NA | 55.6 |
| Randremanana et al. (2016 | NA | Y | Y | Y | U | Y | Y | Y | Y | Y | Y | Y | NA | NA | 90.9 |
| Kabayiza et al. (2014) | Y | Y | U | Y | U | Y | Y | N | Y | NA | NA | NA | NA | NA | 66.7 |
| van Eijk et al. (2010) | Y | N | U | U | U | Y | Y | N | Y | U | Y | NA | Y | Y | 53.8 |
| Chisenga et al. (2018) | Y | Y | U | Y | U | Y | Y | N | Y | NA | NA | NA | NA | NA | 66.7 |
| Hugho et al. (2023) | Y | Y | U | Y | U | Y | Y | Y | Y | NA | NA | NA | NA | NA | 77.8 |
| Randremanana et al. (2012) | Y | Y | U | Y | U | Y | Y | N | Y | NA | NA | NA | NA | NA | 66.7 |
| Platts-Mills et al. (2014) | Y | Y | U | U | U | Y | Y | Y | Y | U | Y | NA | Y | Y | 69.2 |
| McQuade et al. (2020) | Y | Y | U | U | U | Y | Y | Y | Y | Y | Y | NA | Y | Y | 76.9 |
| Terefe et al. (2020) | Y | N | U | Y | U | Y | Y | Y | Y | NA | NA | NA | NA | NA | 66.7 |
| Kiarie et al. (2023) | Y | Y | U | Y | U | Y | Y | Y | Y | NA | NA | NA | NA | NA | 77.8 |
| Chen et al. (2021) | Y | Y | U | Y | U | Y | Y | Y | Y | NA | NA | NA | NA | NA | 77.8 |
| Berendes et al. (2019) | NA | Y | U | Y | U | Y | Y | N | Y | Y | Y | Y | NA | NA | 72.7 |
| Deblais et al. (2023) | Y | Y | U | Y | U | Y | Y | N | Y | NA | NA | NA | NA | NA | 66.7 |
| Budge et al. (2020) | Y | N | U | Y | U | Y | Y | N | Y | NA | NA | NA | NA | NA | 55.6 |
| *Green indicates a* ***low risk of bias;*** *yellow indicates* ***an unclear risk of bias****, and red indicates a* ***high risk of bias****.* | | | | | | | | | | | | | | | |
